# Cost-utility analysis of interferon-free treatments for patients with early-stage genotype 1 hepatitis C virus in Brazil

**DOI:** 10.1590/0037-8682-0594-2019

**Published:** 2020-06-22

**Authors:** Vinicius Lins Ferreira, Leticia Paula Leonart, Maria Lucia Alves Pedroso, Roberto Pontarolo

**Affiliations:** 1 Universidade Federal do Paraná, Programa de Pós-Graduação em Ciências Farmacêuticas, Curitiba, PR, Brasil.; 2 Universidade Federal do Paraná, Hospital de Clínicas, Serviço de Gastroenterologia, Curitiba, PR, Brasil.

**Keywords:** Hepatitis C, Cost-effectiveness analysis, Treatment, Brazil

## Abstract

**INTRODUCTION:**

We conducted a cost-utility analysis of available interferon-free treatments for patients with early-stage genotype 1 chronic hepatitis C based on a Brazilian public health system perspective.

**METHODS:**

A Markov model was derived using a cohort of stage F0-F2 patients treated as recommended by the Brazilian national guidelines.

**RESULTS:**

Glecaprevir plus pibrentasvir was superior to all other treatments, followed by sofosbuvir plus velpatasvir. Sofosbuvir plus daclatasvir was identified as the least cost-effective option.

**CONCLUSIONS:**

The above findings were confirmed via probabilistic sensitivity analysis and the tested scenarios.

Hepatitis C is a viral disease that affects more than 71 million people worldwide and causes almost 400,000 deaths per year, particularly due to its progression to the chronic phase, causing cirrhosis and primary liver cancer^1^. In Brazil, 700,000 patients are estimated to be infected with the hepatitis C virus (HCV), representing nearly 1% of the population^2^.

In the last decade, chronic hepatitis C (CHC) management has undergone intensive modification. In fact, a second generation of direct-acting antivirals (DAAs) was developed to provide more effective, tolerable, and safe treatments for CHC that can be used in combination with other second-generation DAAs to improve treatment response. As a result, the use of first-generation DAAs (i.e., telaprevir and boceprevir) associated with interferon^3^, the gold standard therapy for many years, has been replaced by these newer DAAs.

In 2017, Brazil initiated a schedule to achieve the objectives proposed by the World Health Organization to significantly reduce the number of cases and deaths caused by HCV. This includes extending treatment to all CHC patients (regardless of fibrosis stage), retreatment options, and the incorporation of newer drugs. In 2015, the first DAA options, namely sofosbuvir (SOF), daclatasvir (DAC), and simeprevir, were available via the Brazilian public health system (*Sistema Único de Saúde*, SUS). More recently, other drugs were approved by the Brazilian Health Regulatory Agency and are now offered in SUS, including those for genotype 1 patients [i.e., ledipasvir (LED), glecaprevir (GLE), pibrentasvir (PIB), elbasvir (ELB), grazoprevir (GRA), and velpatasvir (VEL)]^2^.

Although these treatments are linked to high success rates, they are accompanied by relatively high costs. Owing to the remarkable number of HCV patients in Brazil, the availability of many drug options, and the elevated price of hepatitis treatment, a cost-effectiveness analysis is crucial for identifying a strategy that results in the most benefit at a lower cost^4^.

In this context, the aim of this study was to carry out a cost-utility analysis, comparing interferon-free treatments for genotype 1 early-stage CHC patients from a Brazilian public health system perspective.

This study was performed following the Brazilian methodological guidelines regarding health economics evaluations^4^. The software, TreeAge Pro 2016 (TreeAge Software, Inc., Williamstown, MA), was used to generate a decision-analytic Markov model to simulate the natural history of CHC using a cohort of genotype 1, stage F0-F2 patients (non-cirrhotic) treated with the drugs recommended by the Brazilian guidelines (2019) available in SUS (SOF+VEL, SOF+DAC, SOF+LED, ELB+GRA, and GLE+PIB). The drugs used for treatment, recommendations, and posology were obtained from the Brazilian guidelines^2^; treatment combinations without ribavirin were used.

The perspective from SUS, the Brazilian public health system that guarantees full, universal, and free access to the entire population, including patients requiring hepatitis C treatment, was adopted. The initial age of the cohort was 50 years old; this was selected according to the estimated age at diagnosis^5^. Patients were followed until death or 79 years old, whichever came first. A scheme of the model is illustrated in Figure 1. The cycle length was one year, and a within-cycle correction was implemented in each cycle. The cost and outcomes were discounted at a 5% rate.

**FIGURE 1:**
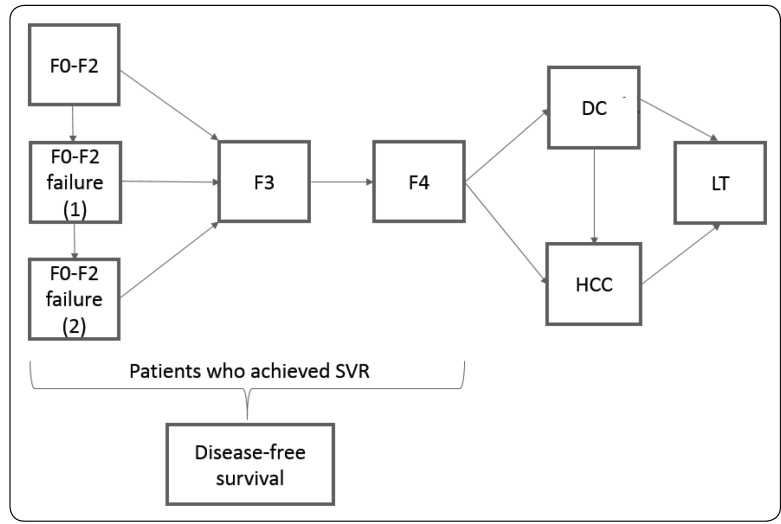
Markov state transition diagram for chronic hepatitis C. *Abbreviations:* **DC:** decompensated cirrhosis; **F:** fibrosis; **HCC:** hepatocellular carcinoma; **LT:** liver transplantation; **SVR:** sustained virological response. *Note:* Patients in stages F0-F4 can achieve SVR and either advance to the disease-free survival stage or to the next stage, or die. The cohort started the model in the F0-F2 stage.

Patients that achieve sustained virological response (SVR) are considered disease-free as natural disease progression to the advanced stages does not occur. However, patients that do not achieve SVR can progress to further stages. Drug efficacy data were obtained from a systematic review^6^ as data regarding some treatments were not available in Brazilian real-word studies (the quality evidence level obtained from this study was considerate high). 

Natural disease progression probabilities were derived from previous economic models. Age-specific mortality rates (death from any cause) were applied throughout the model. Patients in the advanced stages of the disease (i.e., F4, cancer, and transplant stages) had an additional probability of dying due to HCV.

The efficacy outcomes are expressed according to the quality-adjusted life years (QALY). Utility values were obtained from international studies. Each health state in the model was associated with a utility value that represents the patient’s quality of life. Only direct costs were used: drugs, liver transplantation, hepatic cancer, and pre- and post-treatment. 

Values, probabilities, the drug’s efficacy, QALY, costs, and other parameters used in the model are presented in Supplementary Material 1.

The model generated the lifetime costs (currency: reais, R$; treatment costs were collected in 2019) and QALY per patient for each alternative treatment compared. The incremental cost-effectiveness ratio (ICER) would be calculated as the difference in costs divided by the difference in QALY for non-dominated drugs. In Brazil, there is no defined cost-effectiveness threshold; therefore, theoretical values that are one and three-fold greater than the gross domestic product per capita per QALY were adopted (roughly equivalent to R$ 30,000 and 90,000)^2^ ^,^ ^7^.

A probabilistic sensitivity analysis was performed to test the robustness of the model. Probabilistic distributions were applied to each parameter and multiple values were sampled from the results. In this analysis, beta or gamma distributions were selected for each parameter and Monte Carlo analysis was carried out with 5,000 iterations. The results of the simulations are presented in a scatterplot. A cost-effectiveness acceptability graph also revealed the probability of each strategy being the most cost-effective at the defined thresholds (R$ 90,000 and 30,000).

As the Brazilian government usually performs centralized and large-scale purchasing of drugs, purchasing drugs at a discounted price is often possible. Accordingly, we also performed a scenario analysis by applying different discounts on drug price (10%, 20%, 30%, 40%, and 50%).

In another scenario, the short-term schedules for SOF+LED (i.e., 8 weeks) and ELB+GRA (i.e., 12 weeks) were considered. For the other treatments, the schedules were not modified.

Different assumptions were considered for the model: (i) the cohort entered the model at 50 years old and fibrosis stage F0-F2; (ii) 50% of the cohort had genotype 1a while 50% had genotype 1b^8^ (average cost was considered); (iii) 50% of the cohort were treatment naïve while 50% were treatment experienced^8^ (i.e., interferon, ribavirin, first-generation DAA) (average cost was considered); (iv) when treatment failure occurred in the first stage (F0-F2), patients could proceed to the following stages and be treated with a different DAA combination; (v) following treatment failure, any new treatment was administered after 1 year (in the following cycle); (vi) as previously mentioned, patients did not receive ribavirin; (vii) adverse event costs were not considered as most events were not serious and were usually caused by ribavirin^9^; and (viii) treatment discontinuations were not considered due to the low rates^9^. A critical appraisal of this economic study is presented in Supplementary Material 2.

As the SVR rates were similar among the treatment options, the effectiveness results of the model (QALY) were also equal, as expected. GLE+PRI followed by SOF+VEL had the lowest costs, while SOF+DAC had the highest cost. In the base case scenario, GLE+PIB was superior to all other treatments. In fact, it was associated with a lower treatment cost and similar QALY, followed by SOF+VEL. The results of the analysis based on costs and QALY are presented in Table 1 while the cost-effectiveness graph is presented in Supplementary Material 3.

**TABLE 1:** Results of the base case scenario.

Strategy	Cost	QALY
Glecaprevir + pibrentasvir	60679	12.73
Sofosbuvir + velpatasvir	63068	12.73
Sofosbuvir + ledipasvir	103008	12.73
Elbasvir + grazoprevir	123716	12.73
Sofosbuvir + daclatasvir	168226	12.73

*Abbreviations:* **QALY:** quality-adjusted life years.

Although SOF+VEL was dominated by GLE+PIB, the scatterplot shows that both alternatives overlap in most iterations (Figure 2A).

Further, this result was confirmed by the cost-effectiveness as the Monte Carlo acceptability at R$ 90,000 (Figure 2B), where these two DAA combinations presented the highest and similar frequencies (proportion of number of iterations), proved that it is the most cost-effective treatment (56% of interactions for GLE+PIB, 43% for SOF+VEL, 1% for other treatments). Similar results were also obtained at the 30,000-threshold (data not shown).

**FIGURE 2:**
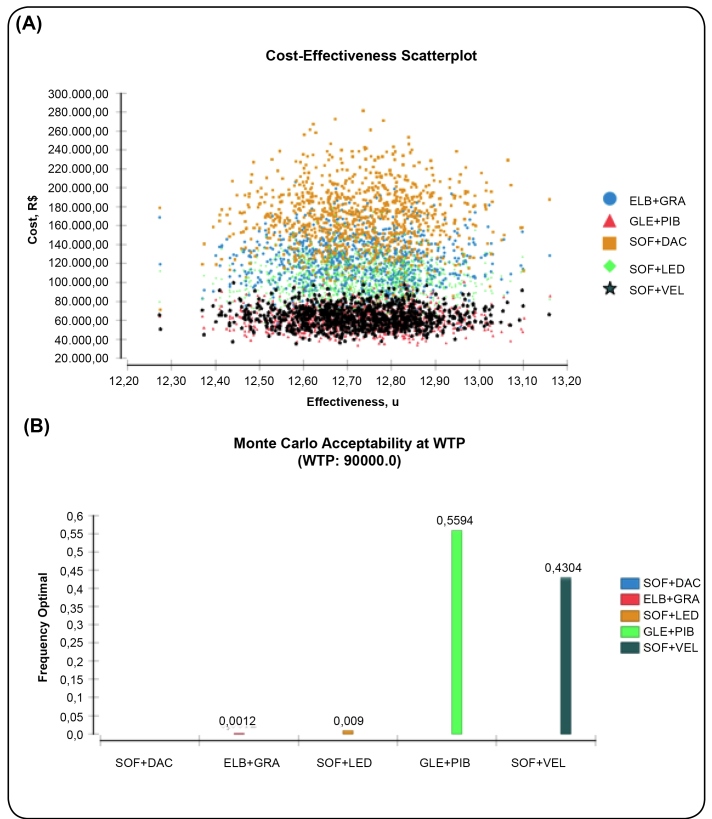
(A) Cost-effectiveness scatterplot and (B) Monte Carlo acceptability at R$ 90,000. *Abbreviations:* DAC: daclatasvir; ELB: elbasvir; GLE: glecaprevir; GRA: grazoprevir; LED: ledipasvir; PIB: pibrentasvir; SOF: sofosbuvir; VEL: velpatasvir; WTP: Willingness to pay; u: utility.

In all scenarios where discounts were applied to the treatment cost (i.e., 10-50%), GLE+PIB continued to have the highest probability of being the most cost-effective treatment at thresholds 30,000 and 90,000 (54-57%), followed by SOF+VEL (41-44%) and SOF+LED (nearly 1%). More details are available in Supplementary Material 4.

The results of the scenario for the short-term schedules with SOF+LED (i.e., 8 weeks) and ELB+GRA (i.e., 12 weeks) were similar to those presented previously. More details are available in Supplementary Material 5.

Second-generation DAAs have become the gold standard treatment for HCV due to their improved safety and effectiveness profiles, leading to withdrawal of prior commonly used treatments (first-generation DAAs and interferon-based therapies). However, second-generation DAAs are well known to be associated with a high treatment cost, thereby warranting the performance of health economic analysis.

Due to the high efficacy rate of the newer DAAs, most patients achieved SVR and were cured in the first cycle (above 95%) in all treatment arms of our model while the remaining patients received another treatment option and achieved SVR in cycle 2. Only few patients (<0.1%) required a third treatment in cycle 3 before they were cured. Therefore, within three treatment cycles, all patients were cured in the early stage of the disease (equivalent to 100%), with no progression to the later stages of cirrhosis, liver transplant, or hepatocellular carcinoma.

In our analysis, SOF+DAC had the worst result among the treatments for genotype 1. As these treatments have a similar efficacy, the cost of the drug had the most impact in the analysis; therefore, SOF+DAC was not a cost-effective option. Unsurprisingly, this combination is no longer recommended for non-cirrhotic patients by the European Association for the Study of the Liver (EASL) nor the American Association for the Study of Liver Diseases/Infectious Diseases Society of America (AASLD/IDSA). In addition, a previous study conducted in Brazil revealed that SOF+DAC may promote the occurrence of mutations in baseline resistance-associated substitutions^10^.

The newest treatment combinations, such as SOF+VEL and GLE+PIB, have lower costs and are thus cost-effective selections in all scenarios. In the probabilistic sensitivity analysis, all iterations demonstrated that both treatments had similar results and were thus regarded as promising treatments. Other studies have also highlighted the cost saving potential of both treatments in scenarios that were either similar or different from our base case^11^ ^-^ ^13^. Furthermore, both treatments enable notable response and are thus good choices.

Recently, the Brazilian government issued a statement regarding the drugs available for the treatment of hepatitis C in SUS based on the cost-minimization criteria. In contrast to our results, this document only recommends the use of SOF+LED for genotype 1 treatment-naïve patients without cirrhosis/renal problems. However, the document still recommends SOF+LED in most situations for treatment-experienced patients. In this document, GLE+PIB is recommended, especially for patients with a creatinine clearance of less than 30 mL/minute. However, as the statement does not include details regarding the costs or other data used in cost minimization, a further analysis could not be carried out^14^.

Adopting the most cost-effective option is important as this guarantees that more patients can gain access to an effective treatment. Further, it enables better resource allocation to other health programs and is an important step in the achievement of the World Health Organization’s plan to eradicate hepatitis by 2030^15^.

Our study had some limitations. First, as some combinations were recently incorporated into the SUS, the real practice costs were not available. As a result, we opted to use the tabulated costs. Second, some costs and parameters, such as adverse events, were not considered and other hypotheses were assumed, which is a normal tactic in any economic analysis.

The present cost-utility analysis revealed that GLE+PIB followed by SOF+VEL was the most cost-effective treatment for CHC patients. Conversely, SOF+DAC was considered to be the least cost-effective option. Accordingly, this combination should be carefully administered in clinical practice.
