# Supplementary material for: Cost-utility analysis of interferon-free treatments for patients with early-stage genotype 1 hepatitis C virus in Brazil
Source: Rev Soc Bras Med Trop. 2020 Jun 22;53:e20190594. doi: 10.1590/0037-8682-0594-2019 (PMC7310368; doi:10.1590/0037-8682-0594-2019)
Supplement: Supplementary file 1 [file 1678-9849-rsbmt-53-e20190594-suppl1.pdf]

Supplementary material 1 - Results of the base case scenario

**Treatments and schedules**			
	**SVR (CI)**	**Distribution**	**Reference**
Sofosbuvir plus daclatasvir, 12 weeks	98% (96–98%)	Beta	[1]
Elbasvir plus grazoprevir, 12 or 16 weeks*	97% (96–98%)	Beta	[1]
Sofosbuvir plus ledipasvir, 8 or 12 weeks**	98% (98–99%)	Beta	[1]
Glecaprevir plus pibrentasvir, 8 weeks	98% (96–100%)	Beta	[1]
Sofosbuvir plus velpatasvir, 12 weeks	96% (95–98%)	Beta	[1]
**Probabilities**			
**Variable**	**Value (SE)**	**Distribution**	**Reference**
F0–F2 to F3	0.03 (0.04)	Beta	[2]
F3 to F4 (CC)	0.1 (0.07)	Beta	[2]
F4 (CC) to F4 (DC)	0.03 (0.01)	Beta	[2]
F4 (CC) to HCC	0.05 (0.01)	Beta	[2]
F4 (DC) to HCC, post SVR	0.01 (0.0075)	Beta	[2]
F4 (DC) to HCC	0.1 (0.01)	Beta	[2]
F4 (DC) to LT	0.11 (0.01)	Beta	[2]
F4 (DC) to death	0.09 (0.01)	Beta	[2]
HCC to LT	0.2 (0.01)	Beta	[2]
HCC to death	0.43 (0.01)	Beta	[2]
LT to death (first year)	0.15 (0.01)	Beta	[2]
LT to death (second and later years)	0.057 (0.01)	Beta	[2]
Cost (R$)			
**Variable**	**Value**	**Distribution**	**Reference**
Pretreatment	440.78	Gamma	[3]
Annual exams, F0–F2 and F3	41.60	Gamma	[3]
Annual exams, F4	249.97	Gamma	[3]
Post-treatment, F3	96.8	Gamma	[3]
Post-treatment, F4	239.1	Gamma	[3]
DC	1440.0	Gamma	[4,5]
HCC	9195.0	Gamma	[3]
LT	70000	Gamma	[3,4,6]
Sofosbuvir + daclatasvir	170868.3	Gamma	[7]
Elbasvir + grazoprevir	1237663	Gamma	[7]
Sofosbuvir + ledipasvir	103192.9	Gamma	[7]
Glecaprevir + pibrentasvir	59268.5	Gamma	[7]
Sofosbuvir + velpatasvir, 12 weeks	59268.5	Gamma	[7]
Retreatment***	103272.9	Gamma	Specialist
Utility			
**Variable**	**Value (SE)**	**Distribution**	**Reference**
F0–F2	0.85 (0.01)	Beta	[8]
F3	0.79 (0.01)	Beta	[8]
F4 CC	0.76 (0.02)	Beta	[8]
F4 DC	0.69 (0.06)	Beta	[8]
HCC	0.67 (0.03)	Beta	[8]
LT (first year)	0.50 (0.07)	Beta	[8]
LT (second and later years)	0.77 (0.05)	Beta	[8]
F0–F2 post SVR	0.92 (0.01)	Beta	[8]
F3 post SVR	0.86 (0.01)	Beta	[8]
F4 post SVR	0.83 (0.01)	Beta	[8]
Death	0	–	–

Treatment alternatives and model parameters (SVR, state transition probabilities, cost, and utility values)
Notes: *16 weeks for genotype 1a and 12 weeks for genotype 1b; **8 weeks for treatment-naïve patients and 12 weeks for treatment-experienced patients ***Re-treatment costs were considered as the mean costs for all treatments (97% SVR for patients that did not achieve SVR).
Abbreviations: CC, compensated cirrhosis; DC, decompensated cirrhosis; HCC, hepatocellular carcinoma; LT, liver transplantation; SVR, sustained virological response

1. Guidelines for the Care and Treatment of Persons Diagnosed with Chronic Hepatitis C Virus Infection [Internet]. Geneva: World Health Organization; 2018 Jul. Web Annex 3.1, Adult hepatitis C virus treatment systematic review. Available from: https://www.nc.

2. Kondili LA, Romano F, Rolli FR, Ruggeri M, Rosato S, Brunetto MR, et al. Modeling cost-effectiveness and health gains of a “universal” versus “prioritized” hepatitis C virus treatment policy in a real-life cohort. Hepatology. 2017 Dec;66(6):1814–25.

3. SIGTAP - Sistema de Gerenciamento da Tabela de Procedimentos, Medicamentos e OPM do SUS. 2019. Available in: http://sigtap.datasus.gov.br/tabela-unificada/app/sec/inicio.jsp.

4. Morais A, Magno LA, Gomide GPM. Impacto da hepatite C sobre o consumo de recursos e custos de pacientescom cirrose hepática no SUS TT - Impact of hepatitis C on resource use and costs of patients with liver cirrhosis in the Brazilian public healthcare system (SUS). J bras econ saúde [Internet]. 2015;7(2). Available from: http://files.bvs.br/upload/S/2175-2095/2015/v7n2/a4975.pdf

5. Borba HHL, Rochau U, Wiens A, Sroczynski G, Siebert U, Ferreira VL, et al. Effectiveness and Cost-Effectiveness of Triple Therapy with Telaprevir and Boceprevir for Chronic Hepatitis C: A Decision Analysis from the Brazilian Public Health System Perspective. Value Heal Reg Issues [Internet]. 2019;20:95–102. Available from: http://www.sciencedirect.com/science/article/pii/S2212109919300573

6. Magno LA, Morais AD. Direct Treatment Costs Of Cirrhosis In The Brazilian Public Health Care System: A 2008-2012 Retrospecteve Analysis. Value Heal [Internet]. 2014 Nov 1;17(7):A364. Available from: https://doi.org/10.1016/j.jval.2014.08.807

7. Agência Nacional de Vigilância Sanitárioa (ANVISA). Câmara de Regulação do Mercado de Medicamentos (CMED). Listas de preços de medicamentos - Preços fábrica e máximos de venda ao governo (PF 0%). 2019. Available from: http://portal.anvisa.gov.br/listas-de-precos

8. Hagan LM, Sulkowski MS, Schinazi RF. Cost analysis of sofosbuvir/ribavirin versus sofosbuvir/simeprevir for genotype 1 hepatitis C virus in interferon-ineligible/intolerant individuals. Hepatology. 2014 Jul;60(1):37–45.
