# Supplementary material for: Cost-utility analysis of interferon-free treatments for patients with early-stage genotype 1 hepatitis C virus in Brazil
Source: Rev Soc Bras Med Trop. 2020 Jun 22;53:e20190594. doi: 10.1590/0037-8682-0594-2019 (PMC7310368; doi:10.1590/0037-8682-0594-2019)
Supplement: Supplementary file 2 [file 1678-9849-rsbmt-53-e20190594-suppl2.pdf]

Supplementary material 2 - Critical appraisal of economic studies methodology

i) Consolidated Health Economic Evaluation Reporting Standards – CHEERS Checklist 1

Reference: Husereau D, Drummond M, Petrou S, et al. Consolidated health economic evaluation reporting standards(CHEERS)—Explanation and elaboration: A report of the ISPOR health economic evaluations publication guidelines good reporting practices task force. Value Health 2013;16:231-50.

Section/item	Item No	Recommendation	Reported on page No/ line No
**Title and abstract**			
Title	1	Identify the study as an economic evaluation or use more specific terms, such as “cost-effectiveness analysis”, and describe the interventions compared.	Page 1
Abstract	2	Provide a structured summary of objectives, perspective, setting, methods (including study design and inputs), results (including base case and uncertainty analyses), and conclusions.	Page 1
**Introduction**			
Background and objectives	3	Provide an explicit statement of the broader context for the study.	Page 2, lines 1 to 24; and page 3, lines 1 and 2.
		Present the study question and its relevance for health policy or practice decisions.	Page 3, lines 3 to 5
**Methods**			
Target population and subgroups	4	Describe characteristics of the base case population and subgroups analysed, including why they were chosen.	Page 3, lines 6 to 24; page 4, lines 1 and 2.
Setting and location	5	State relevant aspects of the system(s) in which the decision(s) need(s) to be made.	Page 3, lines 14 to 16.
Study perspective	6	Describe the perspective of the study and relate this to the costs being evaluated.	Page 3, line 14.
Comparators	7	Describe the interventions or strategies being compared and state why they were chosen.	Page 3, line 11
Time horizon	8	State the time horizon(s) over which costs and consequences are being evaluated and say why appropriate.	Page 4, line 13.
Discount rate	9	Report the choice of discount rate(s) used for costs and outcomes and say why appropriate.	Page 3, line 20.
Choice of health outcomes	10	Describe what outcomes were used as the measure(s) of benefit in the evaluation and their relevance for the type of analysis performed.	Page 4, lines 7 to 10
Measurement of effectiveness	11a	*Single study-based estimates:* Describe fully the design features of the single effectiveness study and why the single study was a sufficient source of clinical effectiveness data.	Not applicable
	11b	*Synthesis-based estimates*: Describe fully the methods used for identification of included studies and synthesis of clinical effectiveness data.	Page 3, lines 23 to 24; and page 4, lines 1 and 2.
Measurement and valuation of preference based outcomes	12	If applicable, describe the population and methods used to elicit preferences for outcomes.	Not applicable

Estimating resources and costs	13a	*Single study-based economic evaluation:* Describe approaches used to estimate resource use associated with the alternative interventions. Describe primary or secondary research methods for valuing each resource item in terms of its unit cost. Describe any adjustments made to approximate to opportunity costs.	Page 4, lines 11 and 12.
	13b	*Model-based economic evaluation:* Describe approaches and data sources used to estimate resource use associated with model health states. Describe primary or secondary research methods for valuing each resource item in terms of its unit cost. Describe any adjustments made to approximate to opportunity costs.	Not applicable
Currency, price date, and conversion	14	Report the dates of the estimated resource quantities and unit costs. Describe methods for adjusting estimated unit costs to the year of reported costs if necessary. Describe methods for converting costs into a common currency base and the exchange rate.	Page 4 line 13
Choice of model	15	Describe and give reasons for the specific type of decision-analytical model used. Providing a figure to show model structure is strongly recommended.	Page 3 line 18
Assumptions	16	Describe all structural or other assumptions underpinning the decision-analytical model.	Page 5 lines 11 and 22
Analytical methods	17	Describe all analytical methods supporting the evaluation. This could include methods for dealing with skewed, missing, or censored data; extrapolation methods; methods for pooling data; approaches to validate or make adjustments (such as half cycle corrections) to a model; and methods for handling population heterogeneity and uncertainty.	Page 4 lines 13 to 19
**Results**			
Study parameters	18	Report the values, ranges, references, and, if used, probability distributions for all parameters. Report reasons or sources for distributions used to represent uncertainty where appropriate. Providing a table to show the input values is strongly recommended.	Supplementary Material 1.
Incremental costs and outcomes	19	For each intervention, report mean values for the main categories of estimated costs and outcomes of interest, as well as mean differences between the comparator groups. If applicable, report incremental cost-effectiveness ratios.	Table 1
Characterising uncertainty	20a	*Single study-based economic evaluation:* Describe the effects of sampling uncertainty for the estimated incremental cost and incremental effectiveness parameters, together with the impact of methodological assumptions (such as discount rate, study perspective).	Not applicable
	20b	*Model-based economic evaluation:* Describe the effects on the results of uncertainty for all input parameters, and uncertainty related to the structure of the model and assumptions.	Page 6, lines 1 to 21
Characterising heterogeneity	21	If applicable, report differences in costs, outcomes, or cost-effectiveness that can be explained by variations between subgroups of patients with different baseline characteristics or other observed variability in effects that are not reducible by more information.	Not applicable
**Discussion**			
Study findings, limitations, generalizability, and current knowledge	22	Summarise key study findings and describe how they support the conclusions reached. Discuss limitations and the generalizability of the findings and how the findings fit with current knowledge.	Pages 6, 7, and 8
**Other**			
Source of funding	23	Describe how the study was funded and the role of the funder in the identification, design, conduct, and reporting of the analysis. Describe other non-monetary sources of support.	Title page (No funding)
Conflicts of interest	24	Describe any potential for conflict of interest of study contributors in accordance with journal policy. In the absence of a journal policy, we recommend authors comply with International Committee of Medical Journal Editors recommendations.	Title page (none)

ii) Critical appraisal guide (Silva et al. 2014)

Reference: Silva EN, Galvão TF, Pereira MG, Silva MT. Estudos de avaliação econômica de tecnologias em saúde: roteiro para análise crítica. Rev Panam Salud Publica. 2014;35(3):219–27.

Respond to each item with "Yes", "No" or "Not clear", except for items 11 to 14, 21, 23, and 24, which should be answered with "Yes", "No", "Not clear" or "Not applicable"

Items	Critical evaluation
**a) Study design**<br>**1. Was the study question properly and clearly presented, and easily answered?**	Yes
**2. Was the target population of the study clearly described?**	Yes
**3. Were the main alternatives included in the study, and was a comprehensive description of the analyzed alternatives provided?**	Yes
**4. Was the model time horizon long enough to reflect the main differences - in cost and health outcome - between the strategies?**	Yes
**5. Was the study perspective informed?**	Yes
**6. Does the study analyze both health costs and outcomes?**	Yes
**7. Was the type of economic evaluation informed?**	Yes
**b) Measurement of health outcomes and costs**<br>**Health Outcomes**<br>**9. Were the health outcome measures clearly described and relevant to the study question?**	Yes
**10. Have the sources of the health outcomes been described and justified and are they in line with the target population?**	Yes
**11. Were the methods and the assumptions used to extrapolate short-term results to final results (medium or long term) described and justified?**	Yes
**12. If health outcomes were derived from clinical trials, does the research protocol reflect what would regularly occur in clinical practice?**	Not applicable
**13. If health outcomes were derived from a systematic review, was the quality of the evidence reported?**	Yes
**14. If the health outcomes were derived from observational studies or assumptions, was the use of this information due to the lack of evidence of better quality?**	Not applicable
**Costs**<br>**15. Were the costs clearly described?**	Yes
**16. Is the measurement of costs in line with the perspective adopted in the study?**	Yes

**17. Was the method adopted to calculate costs described, especially in an adequate manner?**	Yes
**18. Was there information about the currency and the period in which the costs were collected?**	Yes
**19. If the costs were collected in different periods, was there an adjustment for inflation?**	No
**20. Have future costs and outcomes been adjusted using the same discount rate, and was it adequate?**	Yes
**c) Analysis and interpretation of results - Analytical model**<br>**21. Was an analytical model used and was it adequate for the proposed objectives in the study?**	Yes
**22. Do the health states represented in the analytical model reflect the biological process of the disease and the consequences of the technologies used in the research?**	Yes
**23. Has the methodological uncertainty been overcome?**	Yes
**24. Has the structural uncertainty been overcome?**	Yes
**25. Has the uncertainty regarding heterogeneity been overcome?**	Yes
**26. Has the uncertainty regarding the parameters been overcome?**	Yes
**Results**<br>**27. Was the presentation of the study results based on any type of ratio between health costs and outcomes?**	Yes
**28. Was the discussion of the study results broad enough, including the main aspects relevant to patients and the decision makers?**	Yes
**29. Was there information about the internal consistency of the model?**	Yes
**30. Was there information about the external consistency of the model?**	Yes
**d) General information**<br>**31. Was the study's funding source adequately described?**	Yes
**32. Did the authors declare their potential conflicts of interest?**	Yes
**33. Was the study approved by any institution qualified in ethics research?**	No
