# Supplementary figures and images for: Cost-utility analysis of interferon-free treatments for patients with early-stage genotype 1 hepatitis C virus in Brazil
Source: Rev Soc Bras Med Trop. 2020 Jun 22;53:e20190594. doi: 10.1590/0037-8682-0594-2019 (PMC7310368; doi:10.1590/0037-8682-0594-2019)

**Supplementary material 3 – Cost-effectiveness graph for the base case scenario**

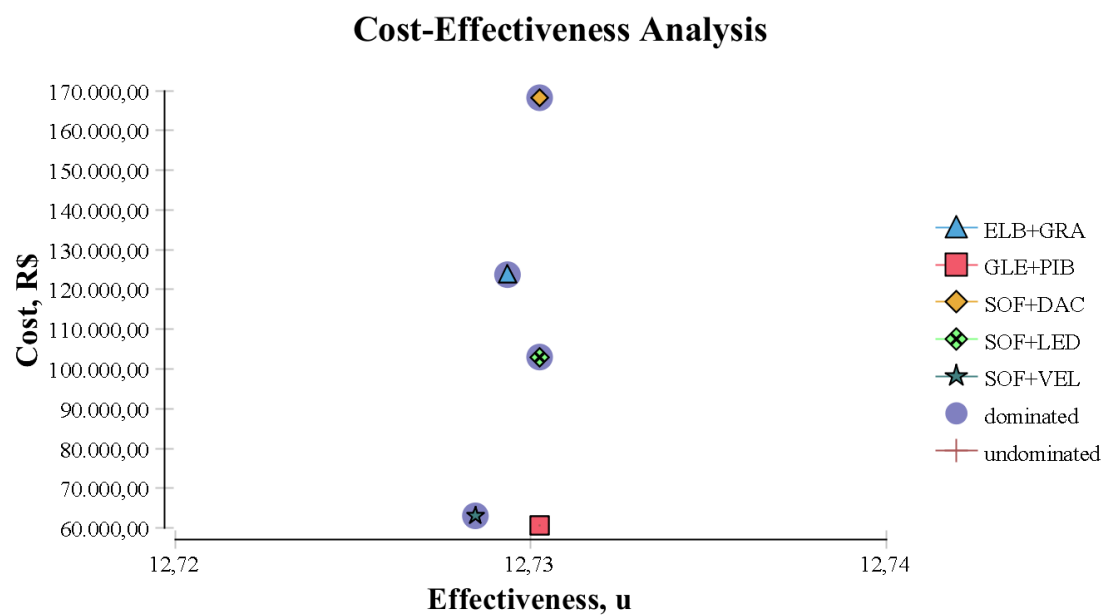

Supplement: Supplementary file 3 [file 1678-9849-rsbmt-53-e20190594-suppl3.pdf]
