# Supplementary material for: Cost-utility analysis of interferon-free treatments for patients with early-stage genotype 1 hepatitis C virus in Brazil
Source: Rev Soc Bras Med Trop. 2020 Jun 22;53:e20190594. doi: 10.1590/0037-8682-0594-2019 (PMC7310368; doi:10.1590/0037-8682-0594-2019)

Supplementary material 4 - Scenario analysis 1: discounts based on drug prices

Strategy	Cost	Incr Cost	Eff	Incr Eff	Incr C/E
**10% discount**					
**GLE+PIB**	54672,1		12,7		
**SOF+VEL**	56823,5	2151,4	12,7	0	DOMINATED
**SOF+LED**	92768,1	38096,1	12,7	0	DOMINATED
**ELB+GRA**	111406,2	56734,2	12,7	0	DOMINATED
**SOF+DAC**	151464,7	96792,6	12,7	0	DOMINATED
**20% discount**					
**GLE+PIB**	48665,0		12,7		
**SOF+VEL**	50578,4	1913,4	12,7	0	DOMINATED
**SOF+LED**	82528,1	33863,2	12,7	0	DOMINATED
**ELB+GRA**	99095,9	50430,9	12,7	0	DOMINATED
**SOF+DAC**	134702,9	86037,9	12,7	0	DOMINATED
**30% discount**					
**GLE+PIB**	42657,9		12,7		
**SOF+VEL**	44333,3	1675,5	12,7	0	DOMINATED
**SOF+LED**	72288,1	29630,3	12,7	0	DOMINATED
**ELB+GRA**	86785,5	44127,6	12,7	0	DOMINATED
**SOF+DAC**	117941,0	75283,2	12,7	0	DOMINATED
**40% discount**					
**GLE+PIB**	36650,8		12,7		
**SOF+VEL**	38088,2	1437,5	12,7	0	DOMINATED
**SOF+LED**	62048,1	25397,4	12,7		DOMINATED
**ELB+GRA**	74475,1	37824,4	12,7	0	DOMINATED
**SOF+DAC**	101179,2	64528,4	12,7	0	DOMINATED
**50% discount**					
**GLE+PIB**	30643,7		12,7		
**SOF+VEL**	31843,1	1199,5	12,7	0	DOMINATED
**SOF+LED**	51808,1	21164,5	12,7	0	DOMINATED
**ELB+GRA**	62164,8	31521,1	12,7	0	DOMINATED
**SOF+DAC**	84417,3	53773,7	12,7	0	DOMINATED

Note:

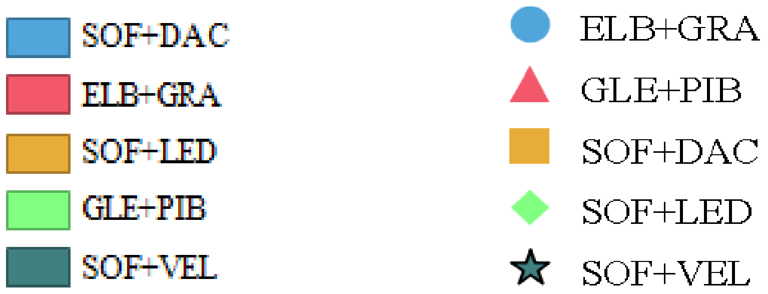

i) 10% discount

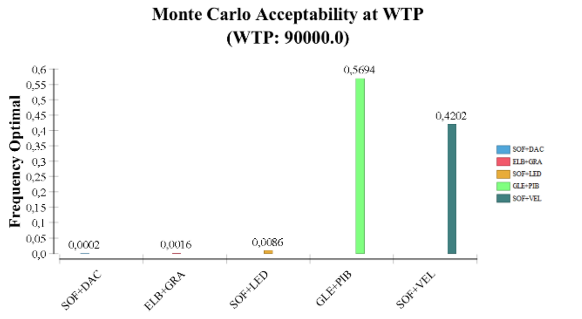

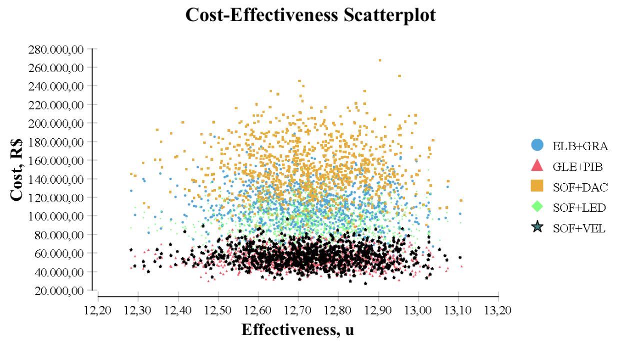

ii) 20% discount

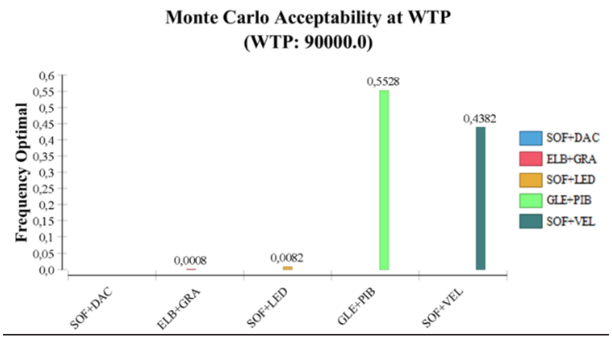

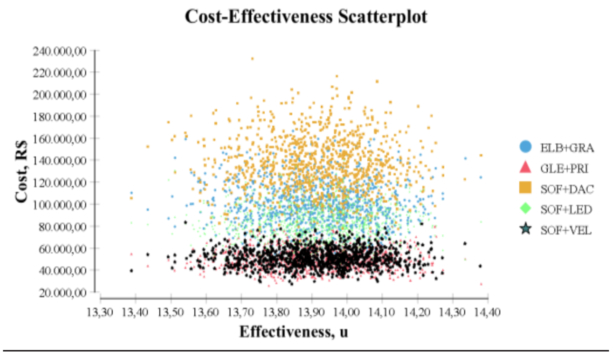

iii) 30% discount

Monte Carlo Acceptability at WTP
(WTP: 90000.0)

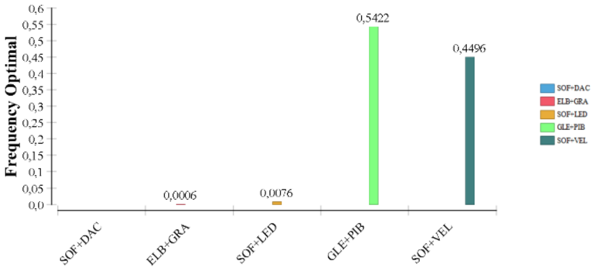

Cost-Effectiveness Scatterplot

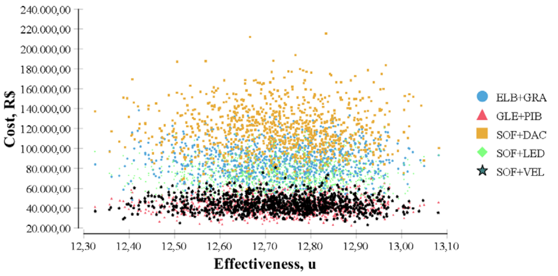

iv) 40% discount

Monte Carlo Acceptability at WTP
(WTP: 90000.0)

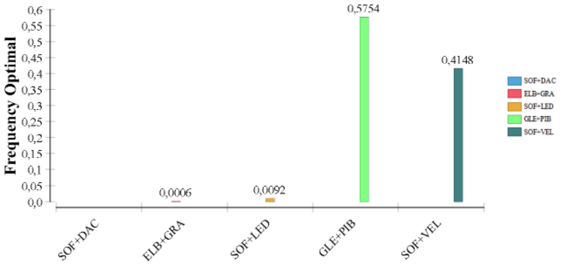

Cost-Effectiveness Scatterplot

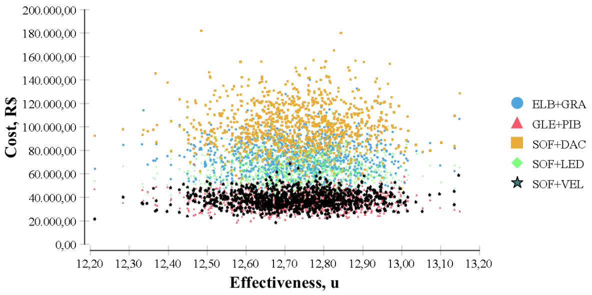

v) 50% discount

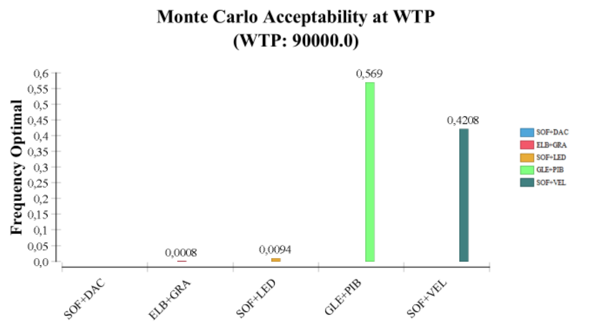

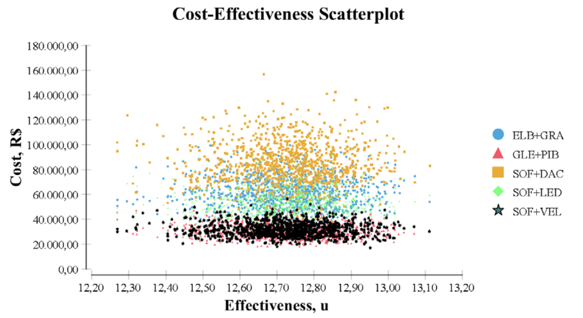

Supplement: Supplementary file 4 [file 1678-9849-rsbmt-53-e20190594-suppl4.pdf]
