# Supplementary material for: Cost-utility analysis of interferon-free treatments for patients with early-stage genotype 1 hepatitis C virus in Brazil
Source: Rev Soc Bras Med Trop. 2020 Jun 22;53:e20190594. doi: 10.1590/0037-8682-0594-2019 (PMC7310368; doi:10.1590/0037-8682-0594-2019)

Supplementary material 5 - Scenario analysis 2: A less expensive schedule of the treatments (SOF + LED for 8 weeks and ELB + GRA for 12 weeks)

Strategy	Cost	Incr Cost	Eff	Incr Eff	Incr C/E
**GLE+PIB**	60679,2	0,0	12,7		
**SOF+VEL**	63068,6	2389,4	12,7	0	DOMINATED
**SOF+LED**	83119,9	22440,7	12,7	0	DOMINATED
**ELB+GRA**	106763,9	46084,7	12,7	0	DOMINATED
**SOF+DAC**	168226,5	107547,4	12,7	0	DOMINATED

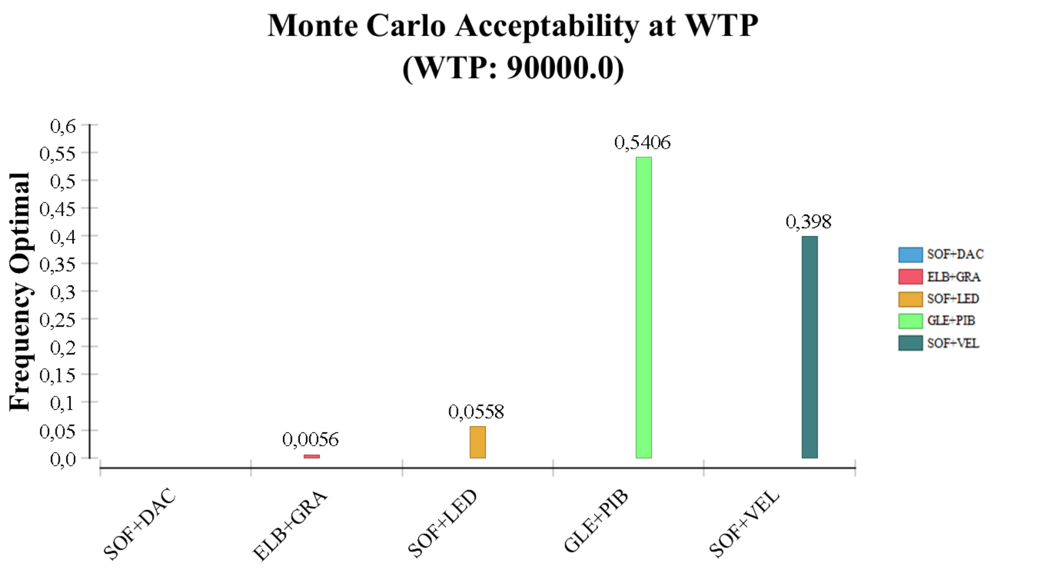

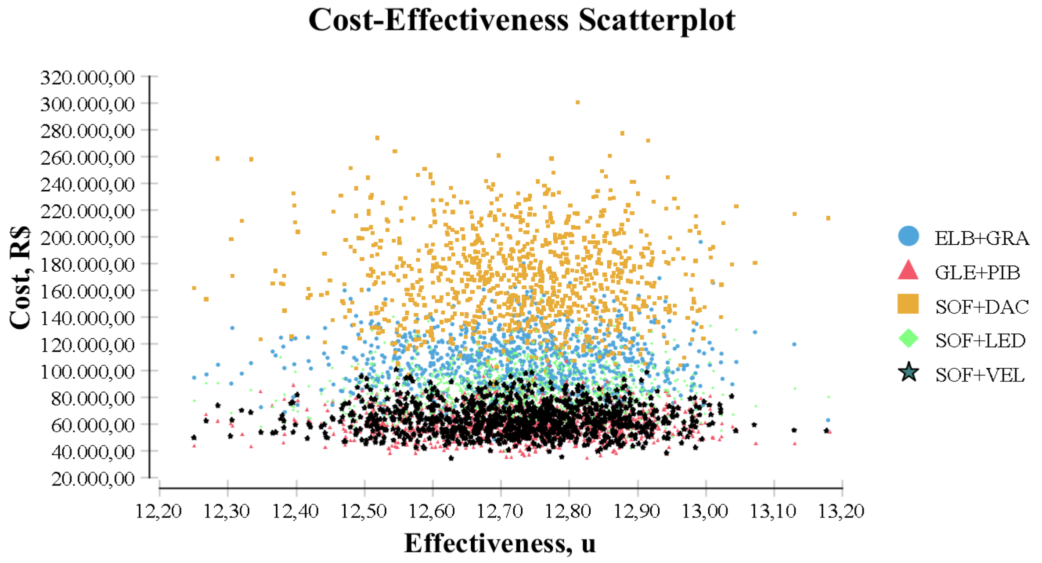

Supplement: Supplementary file 5 [file 1678-9849-rsbmt-53-e20190594-suppl5.pdf]
